# Reduced Intrinsic Connectivity of Amygdala in Adults with Major Depressive Disorder

**DOI:** 10.3389/fpsyt.2014.00017

**Published:** 2014-02-19

**Authors:** Rajamannar Ramasubbu, Nithya Konduru, Filomeno Cortese, Signe Bray, Ismael Gaxiola-Valdez, Bradley Goodyear

**Affiliations:** ^1^Department of Psychiatry, University of Calgary, Calgary, AB, Canada; ^2^Department of Clinical Neuroscience, University of Calgary, Calgary, AB, Canada; ^3^Mathison Centre for Mental Health Research and Education, University of Calgary, Calgary, AB, Canada; ^4^Hotchkiss Brain Institute, University of Calgary, Calgary, AB, Canada; ^5^Department of Radiology, University of Calgary, Calgary, AB, Canada; ^6^Alberta Children’s Hospital Research Institute, Calgary, AB, Canada

**Keywords:** depression, amygdala, fMRI, functional connectivity, neural networks

## Abstract

Imaging studies of major depressive disorder (MDD) have demonstrated enhanced resting-state activity of the amygdala as well as exaggerated reactivity to negative emotional stimuli relative to healthy controls (HCs). However, the abnormalities in the intrinsic connectivity of the amygdala in MDD still remain unclear. As the resting-state activity and functional connectivity (RSFC) reflect fundamental brain processes, we compared the RSFC of the amygdala between unmedicated MDD patients and HCs. Seventy-four subjects, 55 adults meeting the DSM-IV criteria for MDD and 19 HCs, underwent a resting-state 3-T functional magnetic resonance imaging scan. An amygdala seed-based low frequency RSFC map for the whole brain was generated for each group. Compared with HCs, MDD patients showed a wide-spread reduction in the intrinsic connectivity of the amygdala with a variety of brain regions involved in emotional processing and regulation, including the ventrolateral prefrontal cortex, insula, caudate, middle and superior temporal regions, occipital cortex, and cerebellum, as well as increased connectivity with the bilateral temporal poles (*p* < 0.05 corrected). The increase in the intrinsic connectivity of amygdala with the temporal poles was inversely correlated with symptom severity and anxiety scores. Although the directionality of connections between regions cannot be inferred from temporal correlations, the reduced intrinsic connectivity of the amygdala predominantly with regions involved in emotional processing may reflect impaired bottom-up signaling for top-down cortical modulation of limbic regions leading to abnormal affect regulation in MDD.

## Introduction

Major depressive disorder (MDD) is potentially a life-threatening mood disorder with substantial morbidity and mortality (1–3). It is the leading source of disability and disease burden worldwide (4). Despite advances in pharmacological treatment over the last two decades, the burden of depression continues to rise, as 30–40% of patients do not respond to antidepressants (5). As MDD is a complex heterogeneous brain disorder with variations in etiology, symptoms, course, and treatment responsiveness, more research is needed to understand the complexity of the underlying neurobiology of this disorder and to identify potential brain targets for treatment optimization.

A large body of evidence from neuroimaging studies suggests that depression is the result of disruptions of neural circuits encompassing large parts of prefrontal cortex (PFC), limbic, and subcortical structures (6, 7). The current neural models of depression propose emotional dysregulation due to abnormalities in the functioning of the dorsal neural system, also called the cognitive control system, and ventral neural system, also called the emotion appraisal system (8–10). The dorsal system, which comprises the dorsolateral (dl) PFC, dorsomedial (dm) PFC, dorsal (d) anterior cingulate cortex (ACC), and hippocampus, is involved in cognitive processing of emotional input and effortful or voluntary regulation of emotions. The ventral system, which comprises the amygdala, insula, ventral striatum, ventral ACC, and ventral PFC, is crucial for the identification of emotional significance of internal or external stimuli, generation and automatic regulation (regulation without any conscious effort) of affective states, and mediation of autonomic responses depending on stimuli and context resulting in the production of affective states. It has been proposed that increased activity in the ventral neural system and decreased activity in the dorsal neural system may result in a negative bias with predominance in the attention, identification of negative emotions, and other cognitive and vegetative symptoms of depressive disorder (11).

The amygdala is a critical brain region for both bottom-up and top-down processes of emotion generation and regulation (12). It plays a crucial role in bottom-up processes such as emotional perception and encoding affective, motivational and social salience of environmental stimuli, generation of affective state and autonomic responses, directing attention and orienting responses to emotional stimuli, and formation of emotional memory and fear conditioning (13). Studies have identified an increase in amygdala activity in the bottom-up triggering of arousal to potentially threatening stimuli, especially for fearful and angry facial stimuli, even if the observer had no conscious awareness of seeing them (14, 15). Furthermore, the amygdala plays a role in emotional regulation through direct modulation of the medial PFC and ACC, which are considered to be important for top-down regulation of emotion (16–18). Studies in MDD also find abnormally increased hemodynamic and metabolic activity of the amygdala in the resting-state (19, 20) and in response to negative emotional faces presented explicitly, implicitly, or subliminally compared with healthy controls (HCs) corroborating the negative bias hypothesis of depression (21, 22).

As the amygdala interacts extensively within widely distributed cortical–subcortical–limbic circuits, there is increase in number of studies over the last decade examining the functional interactions between the amygdala and other brain regions to understand the disturbances in the functional connections or functional connectivity in amygdala based neural systems in depression. “Functional connectivity” refers to the temporal correlation between the neural activity patterns of anatomically separated brain regions, which is thought to reflect the level of functional communication between the regions (23). Most task-based functional connectivity studies in MDD patients have shown decreased connectivity of the amygdala compared to HCs during negative emotion processing with both dorsal and ventral neural system regions (24–31). However, other studies have shown increased functional connectivity between the amygdala and subgenual cingulate cortex during an emotional face matching task in MDD (26, 32). Although task-related functional connectivity studies provide information on how amygdala connectivity is altered during emotional and cognitive processing, it is crucial to know the abnormalities in the functional connectivity of the amygdala during resting conditions in the absence of any explicit external stimulus or tasks (intrinsic connectivity) to fully understand the dysregulation of amygdala based networks and how this may relate to abnormal response patterns and symptoms in patients with MDD.

In recent years, the resting-state functional magnetic resonance imaging (fMRI) approach has been widely used in psychiatry to probe differences in neural circuits between groups without the potential confounds of task performance and practice/habituation effects. Resting-state data can be processed either by: (1) region-of-interest (ROI) or seed-based analysis, or (2) independent components analysis (ICA) (33). The ROI method is a model-based, hypothesis driven approach, wherein a seed region or ROI is selected *a priori*, and the resulting functional connectivity map is generated from the temporal correlations between the seed region and other brain regions (23, 34). ICA is a model-free or data-driven approach wherein the data of the entire brain is decomposed into spatially distinct clusters (i.e., components) exhibiting similar temporal behavior. This approach is particularly useful for studies with no specific hypothesis as to the brain regions involved (35).

The results of resting-state fMRI studies regarding amygdala connectivity in MDD have been inconsistent. Among six resting-state fMRI studies using model-free ICA, only one study showed deceased bilateral amygdala and left insula resting-state activity and functional connectivity (RSFC) in an affective network (36), whereas the other five studies found no abnormalities in amygdala connectivity (35, 37–40). Among four resting-state fMRI studies using the ROI approach and the amygdala as a seed, one study showed reduced RSFC between ACC and amygdala in non-refractory MDD (41), and another study showed decreased negative intrinsic connectivity between the amygdala seed region and fronto-insular region in MDD (42). However, other two studies did not show altered RSFC of the amygdala either in unipolar depression or major depression compared to HCs (43, 44). Recently, using NAcc as the ROI, Alexopoulos et al. (45) showed reduced RSFC between NAcc and the amygdala in late-life depression. The discrepancy in findings relating to RSFC of amygdala in MDD may be due to small sample sizes, limiting the RSFC analysis of the amygdala only to few predefined target areas, and heterogeneity in clinical characteristics of the study sample. Given the discrepancies in findings and limitations in the previous literature, more studies are needed to investigate the abnormalities of intrinsic connectivity of the amygdala in MDD in a relatively large sample using seed-to-whole brain connectivity analysis to move forward in this field of research.

The main objective of the current study is to examine the abnormalities in the intrinsic functional connections of the amygdala in a large sample of MDD subjects using a seed-based, whole brain functional connectivity analysis of resting-state fMRI data. The seed-based approach is commonly recommended for studies involving a specific hypothesis with *a priori* defined region such as amygdala, because of the simplicity in analysis and interpretation of the results (33). Given that the amygdala has intrinsic connectivity with multiple brain regions (46) and intrinsic connectivity between multiple regions is affected in MDD (33), we preferred to examine the abnormalities of RSFC of amygdala using seed-to-whole brain connectivity analysis. On the basis of the reviewed literature on amygdala and emotional circuitry disturbances in MDD, we predicted that compared to healthy subjects, patients with MDD would show aberrant intrinsic connectivity of amygdala with prefrontal, limbic, and subcortical regions involved in emotional processing and regulation. Based on the findings from resting-state-fMRI studies of amygdala in MDD, we also predicted that MDD patients would show reduced intrinsic connectivity of amygdala with insula, striatum, and ACC compared to healthy subjects.

## Materials and Methods

### Subjects

Fifty-five patients fluent in English and meeting DSM-IV criteria for MDD (47), according to the Structured Clinical Interview for DSM-IV Axis 1 Disorders (48), were recruited through advertisements. The severity of depressive and anxiety symptoms were assessed using the clinician-administered, 17-item Hamilton rating scale for depression (HRSD) and Hamilton anxiety rating scale (HAM-A), respectively (49, 50). Patients were of both genders, right-handed and within the age range of 20–55 years. The Edinburgh Handedness Inventory was used to assess handedness (51). Patients were included in the study if they met the following inclusion criteria: (1) acute episode of MDD of unipolar subtype and a score of 18 or higher on the HRSD, and (2) free of psychotropic medication for a minimum of 3 weeks at recruitment. Exclusion criteria were: (1) axis I disorders such as bipolar disorder, anxiety disorder, or psychosis, (2) history of substance abuse within 6 months of study participation, (3) borderline personality disorder, (4) medical or neurological disorders, (5) severe suicidal symptoms, (6) failure to respond to three trials of antidepressant medication, or (7) contraindications for MRI (metal implants, pregnancy, etc.).

Nineteen HCs, matched by age and gender, were also recruited for the study through advertisements. These participants were screened using the Structured Clinical Interview for DSM-IV Axis I Disorders, non-patient version, to ensure they did not have previous or current Axis I psychiatric disorders (52) or any family history of Axis I disorders by self-report. The demographics of the MDD patients and HC are summarized in Table 1. Ethics approval was obtained from the local review board, and informed consent was obtained from all subjects prior to their participation in the study.

**Table 1:** **Demographic and clinical characteristics for the study sample**.

	MDD (*n* = 55)	Healthy controls (*n* = 19)
Age (years)	36.5 ± 10.41	32.89 ± 9.97
Gender	22 Male/33 female	8 Male/11 female
Education (years)	14.67 ± 2.79	14.89 ± 2.00
HRSD*	21.41 ± 4.18	3.38 ± 1.89
HAM-A*	25.56 ± 5.15	3.63 ± 1.70
Age of onset	24.68 ± 10.41	–
Number of episodes	2.67 ± 3.86	–
Duration of current episode (months)	50.29 ± 63.35	–

MDD, major depression disorder; HRSD, Hamilton rating scale for depression; HAM-A, Hamilton anxiety rating scale. Except for gender, all values are mean ± SD (range), *p < 0.001.

### MRI data acquisition

Images from 63 of the 74 participants were collected using a 3-T General Electric MR scanner (Signa VHi; General Electric Healthcare, Waukesha, WI, USA) equipped with an eight-channel, phased-array head coil. Two resting-state fMRI scans of 230 s (a total of 7 min 40 s) in duration were acquired using a single-shot gradient-recalled echo, echo planar imaging sequence (115 volumes, TR/TE = 2000/30 ms, 24 cm × 24 cm field of view, flip angle = 65°, 30 slices of 4 mm thickness). A T1-weighted structural MRI (TR = 9.2 ms, TE = Minimum, flip angle = 20°, 180 slices of thickness 1 mm) was also acquired for anatomical registration of the fMRI data following analysis. Images of 11 MDD patients were collected using 3 T General Electric MR scanner (Discovery MR 750) using the same parameters and protocol. Participants were required to remain still in the MRI scanner with their eyes open and fixated on a black crosshair at the center of a projection screen. The participants were instructed to relax, not think about anything in particular, and not to fall asleep.

### Data analysis

#### Pre-processing of resting-state fMRI data

All image data were analyzed using the FMRIB Software Library^1^ (FSL). Pre-processing of data included skull/scalp removal, slice timing correction, motion correction, spatial smoothing, and temporal high-pass filtering. MCFLIRT (53) was used to correct for head motion, using rigid-body transformations. Before any multi-session or multi-subject analyses were performed, fMRI data were registered to the anatomical images as well as to the standard Montreal Neurological Institute (MNI) template. This was accomplished using FMRIB’s Linear Image Registration Tool (FLIRT) (53, 54). Slice timing correction was done by using (Hanning-windowed) sinc interpolation to shift each time series by an appropriate fraction of the TR relative to the middle of the TR period. Spatial smoothing was done by applying a Gaussian filter with a full-width at half maximum of 6 mm. Then the images were temporally filtered by 100-s high-pass filter.

Signal fluctuations in the white matter (WM) and cerebrospinal fluid (CSF) predominantly reflect non-neural fluctuations such as scanner instabilities, physiological artifacts, and subject motion (55, 56). These signals are different from the gray matter signal and may introduce temporal coherence that can lead to an overestimation of functional connectivity strength. Hence, these effects were removed by extracting the mean time series of WM and CSF by averaging signal over all voxels within a WM or CSF mask for each time point. The masks of WM and CSF were obtained from each patient’s functional image by selecting voxels within WM and CSF, respectively. The extracted mean WM and CSF time series were then used as temporal covariates during linear regression statistical analysis. A global signal regression was not performed, as this may lead to spurious anti-correlations (57–59).

The left and right amygdala were first located on the standard MNI brain template, using the structures tool available in the FSLView Toolbox. Using FLIRT, these regions were then registered to each participant’s native fMRI image. Average time courses from each of the right and left ROIs were then extracted from the pre-processed resting-state fMRI. Prior to the analysis of images, the synchrony between the left and right amygdala for all participants was determined using the Pearson correlation coefficient, and was compared between MDD patients and HCs (after Fisher *Z* transformation) by Student’s *t*-test.

#### fMRI statistical analysis

Statistical analyses of the imaging data were carried out using fMRI expert analysis (FEAT), which uses a data modeling technique based on the general linear model. The general linear model method used for first-level data (i.e., time series data from a single session) was FMRIB’s improved linear model (FILM). For the current study, a regression model was created to include the left amygdala time series as a predictor and eight nuisance covariates (time series predictors for WM, CSF, and the six motion parameters). The result is an estimate of how well the temporal response of the left amygdala correlates with the temporal signal of each voxel in the brain. This process was repeated again for the right amygdala.

Using FMRIB’s Local Analysis of Mixed Effects (FLAME) in FEAT, we performed higher-level analysis (e.g., analysis across sessions or across subjects or between groups). Given the documented effect of age and sex on resting-state functional connectivity between regions (60–62), these were used as covariates of no-interest in all the group analyses. In addition, to control for scanner-related variations, we used the scanner as the covariate of no-interest in the model. Considering lateralized structural and functional differences (46, 63, 64), statistical parameter maps were generated for the right and left amygdala separately, to determine the mean of each of the patient and control groups, as well as the significant difference between groups. From these analyses, *Z*-statistic images were generated with a statistical threshold of *Z* = 2.3 (*p* = 0.01), and a cluster size threshold of at least 77 contiguous voxels corresponding to a corrected *p*-value of 0.05 as determined by Monte Carlo simulations using AlphaSim^2^.

#### Clinical correlate analysis

To explore the relation between clinical variables and the abnormalities in RSFC of the amygdala in MDD, we computed the Pearson correlation coefficients between the mean *Z* values of clusters that showed significant difference in RSFC contrast maps of MDD versus HC and each of three clinical variables (HRSD score, HAM-A score, and duration of illness since onset). Considering the exploratory nature of the analysis, we used an uncorrected statistical threshold of *p* ≤ 0.05 for each test.

## Results

### Clinical and socio-demographic data

Age, sex, and education were not significantly different between the MDD patient group and the comparison HC group (Table 1). As expected, mean HRSD and HAM-A scores were significantly higher for the MDD group compared with HCs. Furthermore, there were no differences in clinical and socio-demographic characteristics between MDD patients scanned in first scanner and second scanner (Table A1 in Appendix). Although most MDD patients had a HRSD total score of 18 or above at the time of enrollment, the score of 11 patients dropped below 18 on the day of imaging, which was performed 3–7 days after enrollment. All MDD patients, with the exception of three, were previously exposed to antidepressant medications. Four female patients were taking oral contraceptives during the study. Although none of the MDD patients met the criteria for a current anxiety disorder, most of the patients expressed anxiety symptoms (mean HAM-A score 25.56 ± 5.15).

### Differences in RSFC of amygdala between MDD and HC

**Figure 1:**
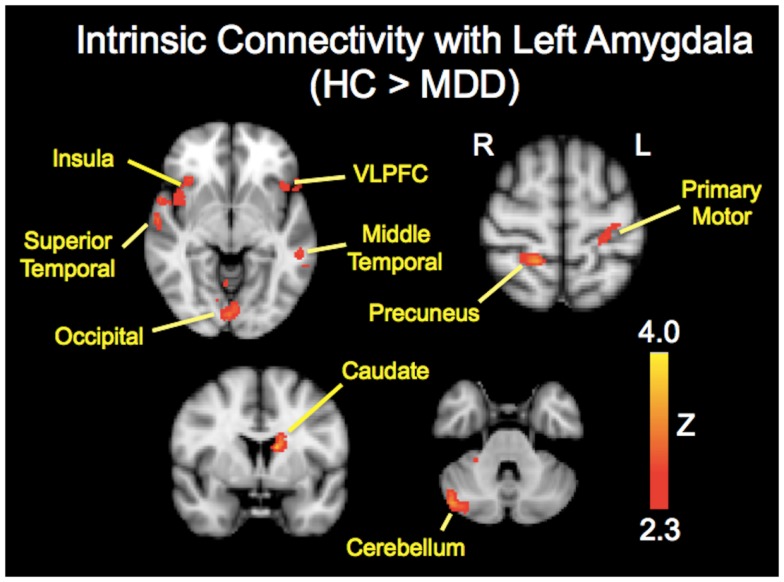
**Brain regions showing reduced resting-state functional connectivity with the left amygdala in depressed patients compared with healthy controls**. Colored areas indicate significant *Z*-scores as determined by fMRI. Statistical threshold of *Z* = 2.3 (*p* = 0.01).

**Figure 2:**
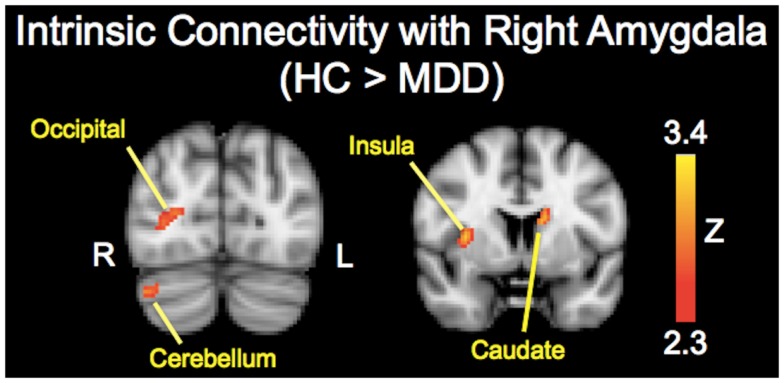
**Brain regions showing reduced resting-state functional connectivity with the right amygdala in depressed patients compared with healthy controls**. Colored areas indicate significant *Z*-scores as determined by fMRI. Statistical threshold of *Z* = 2.3 (*p* = 0.01).

The analysis regarding the synchrony between the left and right amygdala showed that the left amygdala was highly synchronous with the right amygdala for both subject groups (MDD: *r* = 0.87 ± 0.06; controls: *r* = 0.85 ± 0.11), and did not differ significantly (*t* = 0.43, *p* = 0.67). The RSFC analysis demonstrated significantly decreased functional connectivity of both the right and left amygdala with regions including the caudate, insula, occipital regions, and cerebellum (Tables 2 and 3; Figures 1 and 2). The left amygdala exhibited decreased connectivity with additional regions including the vlPFC, precuneus, superior and middle temporal regions, and primary motor cortex in MDD patients compared to HC, suggesting a lateralized pattern of reduced RSFC in MDD. Besides reduced RSFC, both the left and right amygdala exhibited increased connectivity with the contralateral temporal poles in MDD patients, compared to HC. In addition, the left amygdala showed increased connectivity with premotor cortex, while the right amygdala showed increased connectivity with superior temporal region overlapping with inferior frontal cortex, suggesting a differential pattern of connectivity between the right and left amygdala.

**Table 2:** **Brain regions exhibiting a significant difference between patients with MDD and HC in the resting-state functional connectivity with the left amygdala (*p* < 0.05, corrected for multiple comparisons)**.

Region (hemisphere)	BA	Cluster size	MNI coordinate			*Z*-score
			*x*	*y*	*z*	
**HC > MDD**						
VLPFC (L)	44/45	99	−54	18	−2	4.16
Primary motor (L)	4	90	−26	−30	62	2.82
Caudate (L)		131	−12	0	16	3.57
Insula (R)	13	100	40	10	−6	2.98
Precuneus (R)	7	84	16	−44	62	3.3
Middle temporal (L)	21	185	−56	−36	−4	3.33
Middle temporal (R)	21	171	60	−8	−10	3.77
Superior temporal (R)	22	293	58	8	−8	3.17
Occipital cortex (R)	17/18	177	2	−86	−6	3.06
Occipital cortex (L)	17/18	131	−14	−84	−20	3.57
Cerebellum (R)		333	46	−74	−32	3.35
Cerebellum (L)		154	−10	−38	−16	3.99
**MDD > HC**						
Premotor (R)	6	122	38	8	66	4.01
Temporal pole (R)	38	95	34	12	−24	3.29

*BA, Brodmann’s area; HC, healthy controls; MDD, major depression disorder; MNI, Montreal Neurological Institute*.

**Table 3:** **Brain regions exhibiting a significant difference between patients with MDD and HC in the resting-state functional connectivity of the right amygdala (*p* < 0.05, corrected for multiple comparisons)**.

Region (hemisphere)	BA	Cluster size	MNI coordinate			*Z*-score
			*x*	*y*	*z*	
**HC > MDD**						
Insula (R)	13	128	34	2	8	3.13
Caudate (L)		106	−12	0	16	3.05
Cuneus (R)	17	218	24	−82	10	3.14
Occipital/cuneus (L)	17	122	−16	−94	24	3.37
Cerebellum (R)		153	48	−64	−32	3.31
**MDD > HC**						
Temporal pole (L)	38	81	−34	10	−24	2.84
Superior temporal, inferior frontal (L)	21	93	−58	−2	−34	2.96

*BA, Brodmann’s area; HC, healthy controls; MDD, major depression disorder; MNI, Montreal Neurological Institute*.

### Correlation with clinical variables

Given that the left amygdala demonstrated reduced connectivity with more regions than the right amygdala, we performed a correlation analysis between the clinical variables and brain regions with maximum cluster size that showed reduced RSFC with left amygdala. Among the regions that showed increased amygdalar RSFC in MDD, the temporal poles were selected because of bilateral involvement. We found no correlation between clinical variables (HRSD, HAM-A, and illness duration) and left amygdala connectivity with left caudate, right insula, left vlPFC, right precuneus, right cerebellum, and right occipital region. However, we found a significant inverse correlation between the left amygdala–right temporal pole RSFC and the HRSD scores (*r* = −0.31, *p* = 0.02), and HAM-A scores (*r* = −0.46, *p* < 0.001), in MDD patients. Furthermore, the HRSD and HAM-A scores were found to be significantly correlated (*r* = 0.31, *p* = 0.02) probably due to overlapping of anxiety items in both scales and psychopathology of anxiety and depressive symptoms in MDD. This might partly explain the correlation of both test scores with RSFC between left amygdala and right temporal pole. No significant correlation was found between current illness duration and left amygdala intrinsic connectivity with right temporal pole (*r* = −0.05, *p* = 0.74).

## Discussion

In this study, we used seed-based (hypothesis driven) methodology to examine abnormalities in intrinsic connectivity of amygdala in patients with MDD. Confirming our hypothesis, our current data demonstrated reduced intrinsic connectivity of the amygdala with several brain regions implicated in depression including vlPFC, caudate, insula, precuneus, superior temporal gyrus (STG), occipital cortex, and cerebellum in MDD patients compared to HCs. However, we did not find abnormalities in intrinsic connectivity of the amygdala with medial prefrontal regions or ACC. Interestingly, MDD patients showed greater intrinsic connectivity between bilateral amygdala and contralateral temporal poles than healthy subjects and furthermore, intrinsic connectivity of the amygdala with right temporal pole was negatively correlated with depression severity and anxiety scores. Our results provide the first empirical evidence for wide-spread reduction in intrinsic connectivity of the amygdala with several functional networks involved in bottom-up emotional processing and regulation such as the ventral neural system (vlPFC, insula, and caudate), default mode network (precuneus), social cognition (STG), and visual and cerebellar regions.

### Reduced intrinsic connectivity with ventral neural system

It is worth noting that we observed decreased intrinsic connectivity between the amygdala and the right insula using seed-based analysis, which is consistent with previous findings using an ICA approach (36). Similarly, a decrease in intrinsic connectivity (negative connectivity) was observed between the amygdala seed region and fronto-insular region in MDD subjects corroborating our findings of aberrant intrinsic connectivity between amygdala and insula in depression (42). The amygdala and insula have common roles in processing saliency, emotion, and attention and have extensive reciprocal anatomical connections (13, 65, 66). Patients with MDD exhibit hypoactive insula during the resting-state, as well as during emotional task conditions such as negative mood congruent processing, affective switching and in emotional judgment, suggesting biased saliency processing of emotional stimuli in MDD (67, 68). Given the hyperactive amygdala and hypoactive insula in MDD, our findings of reduced amygdala–insula RSFC may suggest disruption in bottom-up saliency processing of negative emotion resulting in reduced self awareness of negative affect feelings, thus leading to negative bias in MDD. Additionally, the reduced amygdala–insula connectivity may hamper the formation of signals in insula to cortical input through the insula–dACC circuit affecting the top-down inhibition of the amygdala. In support of this view, previous work has shown that anterior insula might causally influence activity of dACC, the default mode network and central executive networks (69–71).

The observed attenuated connectivity between the amygdala and caudate highlights the disruptions of limbic–striatal–thalamo-cortical circuits in MDD. Our results are in line with evidence from several neuroimaging studies suggesting the critical involvement of limbic–striatal–thalamo-cortical circuits in the pathophysiology of MDD (72, 73). Both the amygdala and striatum have been implicated in motivational behavior and processing of both positive and negative emotions (13, 74). The amygdala has wide-spread projections to several components of the striatum including the nucleus accumbens and caudate nucleus (75). Thus, the amygdalostriatal anatomical and functional connectivity may be crucial in the formation of signals for cortical input to the premotor, medial, and lateral PFC for motivation and behavioral responses appropriate for the emotional state. Our results showing reduced connectivity between the bilateral amygdala and the left caudate, but not the right caudate, reflects decreased behavioral responses specific to processing negative emotions, as the left striatum is predominantly involved in processing negative emotions and right striatum in processing of positive emotions (68).

Within the prefrontal regions, we found decreased connectivity between amygdala and vlPFC, which has been implicated in processing emotional salience, motivation, and in the regulation of emotional and behavioral responses by modulating emotion-related brain regions such as the amygdala and ventral striatum (76–80). Our results showing reduced connectivity with Broca’s area (left vlPFC BA 44) may reflect impaired interactions between linguistic and cognitive systems (BA 47, 46) that are crucial for emotional evaluation and regulation (81). vlPFC BA 45b is closely related to the orbital frontal network, which functions as part of the ventral neural system for the bottom-up emotional processing and modulation, whereas vlPFC BA 45a has connections and interactions with the dorsal neural system (dlPFC BA 46, 9, 10), which is crucial for top-down emotional regulation (7). Our results showing reduced amygdala connectivity with the vlPFC (BA 44/45) may suggest impaired bottom-up as well as top-down regulation in MDD. This is in line with previous studies reporting decreased activity of vlPFC during voluntary emotional regulation and also decreased effective connectivity between the amygdala and vlPFC in MDD (29, 82).

Contrary to previous studies, we did not find abnormalities in intrinsic connectivity between the amygdala and the medial PFC or ACC (36, 41). The discrepancies between these studies and our study could be ascribed to differences in patient samples and analysis methods. Our patient sample was exposed to medication, and we used a seed-based method, whereas the study by Veer et al. (36) involved depressed patients not exposed to medication and used data-driven ICA methods. Lui et al. (41), using a seed-based approach, showed decreased RSFC between the amygdala and the ACC in non-refractory MDD patients compared to the refractory depression (non-response to two adequate trials) or HCs. Non-refractory MDD patients were drug naïve prior to the study and improved with one or two trials of antidepressant treatment, whereas most of our patients (except three) were exposed to antidepressants and failed to improve with one or two antidepressants trials prior to their participation in this study. Another important source of discrepancy might be related to heterogeneity in amygdala connectivity in MDD (24).

### Hypoconnectivity with posterior regions

The precuneus is a key part of the default mode network, a neural substrate for visual imagery and episodic memory recall, and plays a major role in self-referential processing (34, 83). Taking into account that spontaneous activity in the amygdala negatively predicted activity in regions that are involved in cognitive control of emotions such as lateral PFC, posterior cingulate cortex, and precuneus in healthy subjects (46), and our results in conjunction with previous findings of decreased connectivity within the left amygdala–precuneus region in MDD patients (41) may be indicative of disruptions in bottom-up modulation of emotional processing.

The cerebellum, although underemphasized in the neural model of depression, has been implicated in mood disorders and could play a key role in emotional perception and cognitive processing (84). Electrical stimulation of the cerebellum in animals evoked responses in several brain regions involved in emotional and cognitive processing including PFC, ACC, amygdala, and hippocampus (85). Although it remains unclear whether the cerebellum has a direct inhibitory effect on amygdala responses to negative emotion or whether the amygdala has a modulatory effect on cerebellum, considering the extensive anatomical and functional connections of the cerebellum with prefrontal and limbic regions and the intrinsic connections between centromedial amygdala and cerebellum, our results suggest that amygdala–cerebellar connectivity may play a role in pathophysiology of depression (84).

The amygdala has extensive reciprocal anatomical connections with the visual cortex (86). Emotionally loaded visual stimuli have been shown to increase activation in the amygdala and visual cortex (87). Several imaging studies have demonstrated a possible modulatory role of the amygdala upon visual cortex in processing emotional faces (88, 89) probably through amygdalar efferents to the visual association cortex. Abnormalities in visual cortical regions have also been reported in mood disorders (21, 90). Visual cortex responses to sad faces predicted treatment outcome of antidepressant treatment in MDD (90, 91). Taken together, our results showing attenuated RSFC of amygdala with visual cortical regions (BA 17, 18) suggest disruption in bottom-up processing of emotionally laden visual stimuli in MDD.

### Abnormal connectivity with temporal regions

The STG, temporal pole, and amygdala have been implicated in emotional processing and social cognition such as Theory of Mind (92, 93). Theory of Mind refers to our ability to understand and predict other people’s social behavior by attributing to them independent mental states such as beliefs and desires (94). The STG has been reported to be active during explicit social judgment task whereas the amygdala seems to be involved in rapid automatic implicit judgment of socially salient stimuli (92). Furthermore, MDD patients have also shown decreased STG volume (95), neural activity at rest or in response to sad stimuli (67) suggesting the involvement of STG in the pathophysiology of MDD. Hence our results showing reduced connectivity between the amygdala and the STG may reflect impaired social and emotional processing in MDD. Interestingly, the decreased intrinsic connectivity was observed only with contralateral STG although its functional significance remains unclear. The observed increased intrinsic connectivity with temporal pole may represent compensatory mechanisms, as we found significant negative correlations between RSFC with temporal pole and severity of depression and anxiety scores.

### Right and left amygdala connectivity

Our results showed lateralized patterns of reduced connectivity for the left amygdala in MDD. The left amygdala demonstrated reduced connectivity with more cortical regions (vlPFC, precuneus, and temporal regions) than the right amygdala in MDD. These lateralized abnormalities in amygdala connectivity may reflect the structural and functional differences reported in the literature. The centromedial division of the left amygdala is 25% smaller than the right amygdala (96), and meta-analytic studies have indicated more activation in left than right amygdala during emotional processing (64, 97) probably due to reduced habituation rate and elaborate cognitive and language processing (98, 99). Additionally, there is evidence that top-down responses may modulate only the left amygdala, whereas bottom-up responses may involve both amygdala (12). Consistent with this framework, our results showed reduced connectivity of the left amygdala with cortical regions linked to cognitive and language functioning and top-down regulation. However, the literature regarding the laterality of amygdala dysfunction in MDD is mixed, with results showing on the left, the right, and bilaterally (41, 100, 101). Additional studies are needed to further investigate the question of laterality in intrinsic connectivity of the amygdala in MDD.

### Limitations

There are several limitations that should be considered when interpreting these results. First, unbalanced design may have violated the assumptions underlying group analysis especially homogeneity of variance resulting in statistical error. Hence the results need to be replicated in a balanced design. Second, interscanner and intersubject variability would have affected our results as 11 MDD subjects were scanned in a different scanner. However, we covaried for scanner differences and characteristics of MDD subjects scanned in two scanners were comparable. Third, a seed-based method limits our ability to examine functional connectivity patterns on a whole brain scale beyond the selected regions of interest. Fourth, temporal correlation analysis limits our inference on the directionality or causality of the abnormalities within the regions that are functionally connected. In order to accurately determine the directionality of altered resting-state functional connectivity of the amygdala, we must perform an effective connectivity study using methods such as Granger causality analysis (102–104). Furthermore, the observed reduced intrinsic connectivity of the amygdala could be a maladaptive response to a hyperactive amygdala. Future studies should take an integrative approach to examine the relation between resting-state activity and connectivity and how these may relate to the psychopathology of depression and progression of illness or clinical staging from vulnerability to refractory depression. Fifth, although patients were unmedicated at the time of imaging, prior exposure to long-term antidepressant medication might have an enduring effect on RSFC. Our results therefore need to be confirmed in drug naïve MDD patients. Sixth, since the participants in this study had moderate depression, our findings may not generalize to patients with severe depression. Seventh, the amygdala has three subdivisions (i.e., laterobasal, centromedial, and superficial) with distinct functional connections with limbic and cortical regions (46); we did not examine each division separately. However, our results indicate abnormalities in intrinsic connectivity of all three subdivisions of the amygdala in MDD patients given that in healthy subjects, resting-state activity in the laterobasal, centromedial, superficial subdivisions predicted activity in temporal/frontal regions, striatum/cerebellum, and limbic structures, respectively (46). Eighth, all the observed abnormalities may not reflect psychopathology of depression (state marker) as some may be related to vulnerability for depression (trait marker). Longitudinal prospective studies with treatment intervention are needed to determine state and trait related abnormalities in the intrinsic connectivity of the amygdala in MDD. Finally, it is possible that the group differences in worrying and anxiety at the time of scanning may have influenced our results as the anxiety state may increase resting-state activity in amygdala. However, it remains unclear whether increase in resting-state activity of amygdala may result in decrease in intrinsic connectivity or both are independent process. Future studies should address this question by investigating resting-state amygdala activity and FC in the same cohort.

## Conclusion

The present study extended the findings of previous studies by investigating altered RSFC of the amygdala with the entire brain using a seed-based approach in MDD patients compared to HCs. Our study suggests abnormally decreased intrinsic connectivity between the amygdala and regions of the ventral neural system (vlPFC, insula, and caudate), which are known to be involved in bottom-up modulation of emotional processing and regions involved in self and social cognition (precuneus and STG) as well as posterior regions (occipital and cerebellum) implicated in emotional and cognitive processing. This abnormally decreased intrinsic connectivity of the amygdala could provide a neural network basis for a dysregulated, hyperactive amygdala, and for depressive symptoms including negative bias, impaired social cognition, and diminished motivational and behavioral responses in major depression.

## Conflict of Interest Statement

The authors declare that the research was conducted in the absence of any commercial or financial relationships that could be construed as a potential conflict of interest.

## Footnotes

^1^http://www.fmrib.ox.ac.uk/fsl/

^2^http://afni.nimh.nih.gov/afni/doc/manual/AlphaSim

## Appendix

**Table A1:** **Characteristics of MDD subjects scanned in two scanners**.

	Scanner 1 (*n* = 44)	Scanner 2 (*n* = 11)	*p*-Value
Age (years)	36.84 ± 10.34	36.09 ± 10.45	0.85
Gender	16 Male/28 female	6 Male/5 female	0.32
Education (years)	14.67 ± 2.79	14.89 ± 2.00	0.15
HRSD	21.50 ± 3.76	20.55 ± 4.32	0.41
HAM-A	23.93 ± 4.62	21.82 ± 5.23	0.19
Age of onset	24.73 ± 9.85	26.55 ± 13.74	0.62
Number of episodes	2.98 ± 4.22	1.64 ± 1.80	0.32
Duration of current episode (months)	50.00 ± 64.26	25.36 ± 51.60	0.16

*MDD, major depression disorder; HRSD, Hamilton rating scale for depression; HAM-A, Hamilton anxiety rating scale*.
